# The effects of prenatal HIV exposure on language functioning in Kenyan children: establishing an evaluative framework

**DOI:** 10.1186/s13104-016-2264-3

**Published:** 2016-10-12

**Authors:** K. J. Alcock, Amina Abubakar, Charles R. Newton, Penny Holding

**Affiliations:** 1 Department of Psychology, Fylde College, Lancaster University, Lancaster, LA1 4YF UK; 2 Centre for Geographic Medicine Research-Coast, KEMRI, Kilifi, Kenya; 3 Department of Public Health, Pwani University, Kilifi, Kenya; 4 Department of Psychiatry, University of Oxford, Oxford, UK; 5 Saving Brains Platform Team, Mombasa, Kenya

**Keywords:** HIV, Language, Africa, Children

## Abstract

**Background:**

HIV infection has been associated with impaired language development in prenatally exposed children. Although most of the burden of HIV occurs in sub-Saharan Africa, there have not been any comprehensive studies of HIV exposure on multiple aspects of language development using instruments appropriate for the population.

**Methods:**

We compared language development in children exposed to HIV in utero to community controls (N = 262, 8–30 months) in rural Kenya, using locally adapted and validated communicative development inventories.

**Results:**

The mean score of the younger HIV-exposed uninfected infants (8–15 months) was not significantly below that of the controls; however older HIV-exposed uninfected children had significantly poorer language scores, with HIV positive children scoring more poorly than community controls, on several measures.

**Conclusions:**

Our preliminary data indicates that HIV infection is associated with impaired early language development, and that the methodology developed would be responsive to a more detailed investigation of the variability in outcome amongst children exposed to HIV, irrespective of their infection status.

## Background

Despite the fact that the majority of HIV exposed children live in sub-Saharan Africa (SSA), current knowledge of the effects of HIV on language functioning in the region is scant. Limited conclusions can be drawn from the four studies from SSA that have looked at the effects [1–4], primarily because of the restricted scope of the language measures used. The current study aims to evaluate a methodology, a locally developed and validated communicative development inventory (CDI), to enable a more detailed analysis of language functioning among HIV exposed children at an early age, both infected and uninfected.

Important considerations in evaluating language acquisition are the continuities and discontinuities in the development of communicative abilities. Children with early delays in gesture and comprehension, for example, are at increased risk of delayed spoken language and of a continued language delay [5, 6]. A common association of early language delay is impaired grammatical development [7]. Evidence from Western countries indicates that HIV infection is associated with impairments of both expressive and receptive language in young children [8, 9], with a greater difficulty in expressive language functions [8]. In addition, the gap between control populations and children infected with HIV appears to widen as children grow older [10]. A better understanding of both the effect of exposure to infection, and the potential pathways to risk, will be provided by more detailed contextually validated measurement tools.

Language tests, perhaps even more than other neuro-cognitive assessments, require careful adaptation and evaluation before their reliability and validity can be inferred. One of the difficulties in adapting any developmental assessment for a new situation is that of cultural variance—items may be inappropriate or performance may be poor due to unfamiliarity with materials or vocabulary [11, 12]. Additional issues with the adaptation of linguistic assessments stem from strongly culture-bound linguistic behaviours, reluctance of young children to participate in language assessments, and the large differences in grammatical and lexical structure found between languages [13].

We use the CDI to assess different aspects of language functioning, gestures, comprehension, production and early syntactic development, to test the following hypotheses:

- Both infected and uninfected children exposed to HIV will have poorer language development than community controls [14];
- Effects should be observable as early as 8 months of age; with the strength of the effect becoming amplified with age [10];
- More challenging aspects of language will show larger delays i.e. there will be larger delays in language production than in gesture or comprehension in younger children, and larger delays in grammatical abilities than in vocabulary production in older children [15].

## Methods

### Study site

The study was carried out in Kilifi, a largely rural district on the Kenyan coast. The majority of the population belongs to the Mijikenda ethnic group. Of the Mijikenda languages Kigiriama is the most commonly spoken language in Kilifi district. Approximately two-thirds (66.8 %) of the population in Kilifi lives below the poverty line [16]. The HIV prevalence is estimated at 11 % in the general population and 9 % among pregnant women [17]. Anti-retroviral treatment was not routinely available at the time the study was undertaken (2004–2005).

### Participants

Mother–child dyads were eligible for recruitment if (a) the child was aged 8–30 months, (b) both parents spoke Kigiriama as their primary language and (c) parents provided informed consent. Three groups of children were identified: (i) HIV infected children of HIV positive mothers, whose own status was verified by a positive HIV antibody test if older than 18 months, or polymerase chain reaction (PCR) positive if younger than 18 months; (ii) HIV exposed group of children born to HIV positive mothers who, following similar testing, were found to be HIV negative; and (iii) the community controls.

#### Recruitment of children of HIV positive mothers

The attending physician identified and approached all mothers with eligible children visiting the Family Health Clinic during the study period (September 2005 and June 2006). A total of 52 families were approached, 49 were recruited into the study, and 3 families declined.

#### Recruitment of community controls

Children in this group were recruited as part of a larger study developing reference data for measures of early development appropriate for use in East Africa [18]. Children qualified for inclusion if they were residents of Kilifi District and were reported not to suffer from any chronic disease or infection. We did not test systematically for the HIV status of community control children because ad hoc testing was locally unacceptable, and no treatment was available at the time should a child be identified as HIV positive.

Based on the prevalence in the community we estimated that in this population of 262 randomly selected children, approximately 24 children may have been prenatally exposed. There was an expected mother–child transmission rate of 25–40 % suggesting that only 6–10 of the children may have been HIV positive. This is likely to be an overestimate as it does not take into account the high mortality rates (up to 50 %) by age two in this population pre-ARV era. This potential inclusion of a very small percentage of HIV positive children in the control group may reduce the magnitude of the impact of HIV on language but would not invalidate our findings.

### Measures

#### Language measures

Language development was assessed using two age-specific versions of local adaptations of the MacArthur-Bates CDI. The CDI is a parent report measure. The use of parent report measures of language is best practice in studies of health and cognitive development, especially in developing countries where infants are likely to find direct assessment of their abilities challenging, and are unlikely to respond well to unfamiliar testers [11, 19, 20].

Local adaptations in Kigiriama were developed, based upon close observations of the development of gesture, vocabulary and syntax in the local community, and linguistic descriptions of the language, not simply through direct translations of the original protocol. As most parents in the community are illiterate, the inventories were administered in interview format. Test construction and validation have been described in detail elsewhere [21]. In summary, the instrument has excellent psychometric properties with alphas for the subscales and full scales ranging from .750 to .987. Test–retest reliability was indicated by significant correlations between scores at two time points 1 week apart. Additionally, sensitivity to age was observed indicating that the CDI forms picked up expected maturational changes [21]. The CDI has different versions for different stages of language acquisition. The Words and Gestures version (*Maneno na Ishara*) was administered to parents of children aged 8–15 months. As in the original CDI the *Maneno na Ishara* asks parents about their child’s comprehension and production of single words as well as the use of simple gestures. An additional item, asking about the use of word combinations, was added, suggested by pilot study responses as appropriate for children in the target community. Children receive scores for the total number of words they comprehend but do not produce, the total number they produce, and the total number of gestures they use. The sum of these creates a total language score. Overall children were assessed with this instrument (112 community controls, 6 HIV exposed children and 5 HIV infected children).

The words and sentences version (*Maneno na Sentensi*) was administered to parents of children aged 16 to 30 months. *Maneno na Sentensi* assesses production of single words, grammatical morphemes, and word combinations. Scores are given for the total number of words produced and a score for grammatical complexity. The sum of these scores creates a total language score. Overall 139 children were assessed with this version (123 community controls, 6 HIV exposed children and 10 HIV infected children).

#### Maternal education

A dichotomous schooling variable (schooled vs. unschooled) was created. “Schooled” was defined as having attended at least 1 year of formal schooling.

#### Children’s anthropometrics

Children were weighed at least three times to achieve consistent results within one decimal place. Weight-for-age was computed using WHO Anthro 2005 [22]. Being underweight was defined as having a weight-for-age score less than −2.00 SD to the WHO references scores.

### Procedure

Children and their primary caregivers were seen at home after an initial visit to introduce the study, obtain consent and gather information on mothers’ educational levels. Recruitment was carried out by research assistants who were not involved in interviewing, and interviewers were not informed of families’ HIV status. Teams of two experienced interviewers, trained prior to data collection, carried out the language inventory interview and weighed the children. The interviewers were not informed of the children’s status, or group membership. However, due to the physical condition of some children and family members, in some instances interviewers may have become aware of the children’s HIV exposure.

#### Ethics

The Kenya Medical Research Institute National Scientific and Ethical Committees approved the study (SCC No: 832). Informed consent was obtained from all families and guardians of study participants.

## Results

### Sample characteristics and variable distributions

Table 1 presents a detailed summary of the characteristics of the children and their mothers. No significant differences were found between groups in these background characteristics.

**Table 1:** Means (and standard deviations) of the various background variables

Variables	Community controls	HIV exposed	HIV infected	P
Sample size (females)	261 (129)	14 (6)	18 (7)	
Mean children’s age months (SD)	16.90 (7.02)	16.73 (7.85)	18.10 (8.58)	.78
Mean maternal education (SD)	3.44 (3.57)	4.21 (4.44)	4.28 (3.93)	.07
Mean children’s weight-for-age (SD)	−1.18 (1.11)	−1.29 (.86)	−1.79 (1.44)	.08

The language scores were skewed so that analyses reported here were carried out on transformed data. Tables of results report raw scores however.

### Testing for covariates

Bivariate correlations among background variables and languages scores were calculated to test for potential covariates that might be related to language development, independent of HIV status. Results showed that child’s age was the only variable that was significantly correlated with language development r (293) = .79, p < .001. A *t* test indicated that there were no significant differences in the language performance of boys and girls (M = 161.60, SD = 170.08 and M = 153.00, SD = 146, t (289) = .43 p = .67 respectively). Additionally, language performance was not related to nutritional status (weight-for-age) or maternal education. Given the above results, only age was included as a covariate in all subsequent analysis.

### Effects on language scores

Among younger children (aged 8–15 months), the HIV infected children obtained lower mean scores compared to the community control group in all aspects of language investigated although these differences were not statistically significant (see Table 2). The HIV infected children produced and comprehended fewer words, used fewer gestures and were less likely to combine words. An ANOVA with age as a covariate indicated that neither of these reached significance (F_2, 117_ = .45, p = .64) for the total score. Similar patterns of results were observed in subscale measures (gesture; F_2, 117_ = 1.46, p = .24; comprehension: F _2, 119_ = .14, p = .87; Production: F_2, 119_ = .02, p = .99).

**Table 2:** Age adjusted means and standard error of language scores for children aged 8–15 months

Variables	Community controls	HIV exposed	HIV infected	Maximum possible score
Total language score	95.35 (5.34)	95.78 (23.24)	75.17 (25.45)	365
Comprehension	73.94 (4.50)	75.77 (19.86)	60.04 (21.70)	292
Production	8.44 (1.11)	6.53 (4.88)	4.07 (5.32)	292
Gestures	21.34 (.99)	20.08 (4.30)	15.12 (4.71)	73

Among older children (aged 16–30 months) both HIV groups (infected and uninfected) obtained lower mean scores compared to the Community Control group in all areas of language investigated. Furthermore an ANOVA with age as a covariate indicated that there was a significant main effect of HIV status on total language scores at this age (F_2, 132_ = 6.78 p = .002, η^2^ = .09). The partial eta squared indicates a moderate effect size. Post-hoc analyses revealed that the HIV infected group performed significantly more poorly than the community controls (mean difference = 3.33, std. error = 1.25, p = .009, Cohen’s d = .71). The scores of the exposed, uninfected, children placed them in between the two groups (see Table 3).

**Table 3:** Age adjusted means and standard error of language scores for children aged 16–30 months

Variables	Community controls	HIV exposed	HIV infected	Maximum possible score
Total language	255.08 (11.72)	186.4 (64.53)	164.44 (41.06)	761
Production	196.58 (11.07)	133.45 (60.96)	111.27 (38.79)	702
Grammatical complexity	8.50 (.92)	2.98 (5.09)	3.16 (3.23)	59

Similarly, a significant effect of HIV status was observed on the production subscale (F_2, 131_ = 3.36, p = .004 η^2^ = .08). The partial eta squared indicates a moderate effect size. Post-hoc analyses revealed that the HIV infected group performed significantly more poorly than the community controls (mean difference = 4.14, std. error = 1.44, p = .005, d = .71).

Finally, a significant effect of HIV status was observed on the total score for grammatical complexity and word combining (F_2, 130_ = 4.208, p = .017 η^2^ = .06). The partial eta square indicates a moderate effect size. Here post hoc analyses revealed again that the HIV infected group performed significantly more poorly than the community controls (mean difference = 1.14, std. error = .493, p = .022, d = .53).

The scores of the children in the HIV exposed, uninfected, group did not differ significantly from either of the other two groups. However the effect size for the differences between this group and the community controls was moderate (d = .54, .52 and .55 respectively for total language score, vocabulary production, and grammatical complexity).

## Discussion

Our results indicate that the CDI provided a methodology that was responsive to the effect of prenatal HIV exposure, and, despite the small sample sizes available for this study, indicated a negative impact of HIV exposure on early language development. Our results provide some support for each of the stated hypotheses, and enable us to be more confident that this tool is appropriately measuring the impact of HIV on language development.

Infected children had a generalised delay across all areas on language functioning measured. For the exposed, uninfected children, their relative pattern of development appeared to change over time. Early in language development, this group demonstrated a performance that was similar to, if not superior to, that of the community controls. At later stages, as language becomes more complex, the performance of the uninfected children failed to develop as quickly, resembling more closely that of the infected children. HIV infected children in contrast consistently obtained lower mean scores compared to the community controls. However, significant differences between group scores could only be seen after 16 months of age.

Results from other studies are variable, suggesting potentially different pathways to language impairment, by exposure and by context. In the US, McNeilly et al. observed that HIV exposed children had an increased risk of impaired language functioning compared to the HIV infected groups [9]. Furthermore studies from Western settings also reported that language impairments in the HIV population can be seen early in language development, and are apparent before impairments in other functional domains [9, 10].

Similarly to our findings, Van Rie et al. in the Democratic Republic of the Congo found that HIV exposed children were more likely to score in the “delayed” range than control children on expressive language, though less likely to be classified as “delayed” than HIV infected children [3]. Other studies from Africa did not also find significant differences between groups before children’s second birthday [1–4]. Based on the premise that these measures, specifically designed for the population, may be more sensitive, we expected to find an early effect of HIV exposure on language acquisition, but we did not find this result. Our language measure was responsive to maturational effects, thus we are unable to currently conclude whether the lack of a significant effect at this younger age is in line with previous results in Africa, or may have been due to the small sample size.

The differences observed in our sample between the three groups, as well as the wide variability in performance of HIV infected children, suggest that a variety of mechanisms contribute to language risk or resilience, and that the impact of these mechanisms may be greater at older ages, or may act cumulatively over time [23]. The potential pathways are multiple, although it requires a larger sample to investigate in detail the relative contribution of the sources of this variability in performance. First, vertical transmission may lead to subsequent HIV related damage to the central nervous system [24, 25]. Second, compromise to prenatal environment may lead to higher incidences of low-birth weight and prematurity factors [26, 27] which have been associated with poor developmental outcomes. Third, compromise of the postnatal home environment may act by limiting stimulation through suboptimal parenting behavior [28]. Specific mechanisms for this last pathway might include exposure to stressors such as poor maternal mental health, maternal chronic illness, or constrained economic circumstances leading to restricted resources such as fewer toys and less opportunity for stimulation [28].

A key limitation of our study, as we have stated, is the small sample size of our two at risk groups. While this may have limited our ability to derive statistical significance, our intention was to identify a methodology that might enable a closer scrutiny of patterns of development, and this we achieved. In subsequent applications of this methodology we intend to explore the possible impact of exposure to stressful and a non-stimulating home environment on child’s language development with the additional inclusion of a direct measure of these influences.

## Conclusions

The language development of children in tropical regions, especially SSA, is vulnerable to early brain insults that result from exposure to diseases such as cerebral malaria, meningitis, and sickle cell [29, 30]. Our study is strongly supportive of the potential risk that HIV poses to language development. The sources of variability in outcome warrants further evaluation.
